# Recessive missense *LAMP3* variant associated with defect in lamellar body biogenesis and fatal neonatal interstitial lung disease in dogs

**DOI:** 10.1371/journal.pgen.1008651

**Published:** 2020-03-09

**Authors:** Kati J. Dillard, Matthias Ochs, Julia E. Niskanen, Meharji Arumilli, Jonas Donner, Kaisa Kyöstilä, Marjo K. Hytönen, Marjukka Anttila, Hannes Lohi

**Affiliations:** 1 Department of Veterinary Biosciences, University of Helsinki, Helsinki, Finland; 2 Department of Medical and Clinical Genetics, University of Helsinki, Helsinki, Finland; 3 Folkhälsan Research Center, Helsinki, Finland; 4 Veterinary Bacteriology and Pathology Research Unit, Finnish Food Authority, Helsinki, Finland; 5 Institute of Functional and Applied Anatomy, Hannover Medical School, Hannover, Germany; 6 Institute of Functional Anatomy, Charité - Universitaetsmedizin Berlin, Berlin, Germany; 7 German Center for Lung Research (DZL), Berlin, Germany; 8 Genoscoper Laboratories Ltd (Wisdom Health), Helsinki, Finland; HudsonAlpha Institute for Biotechnology, UNITED STATES

## Abstract

Neonatal interstitial lung diseases due to abnormal surfactant biogenesis are rare in humans and have never been reported as a spontaneous disorder in animals. We describe here a novel lung disorder in Airedale Terrier (AT) dogs with clinical symptoms and pathology similar to the most severe neonatal forms of human surfactant deficiency. Lethal hypoxic respiratory distress and failure occurred within the first days or weeks of life in the affected puppies. Transmission electron microscopy of the affected lungs revealed maturation arrest in the formation of lamellar bodies (LBs) in the alveolar epithelial type II (AECII) cells. The secretory organelles were small and contained fewer lamellae, often in combination with small vesicles surrounded by an occasionally disrupted common limiting membrane. A combined approach of genome-wide association study and whole exome sequencing identified a recessive variant, c.1159G>A, p.(E387K), in *LAMP3*, a limiting membrane protein of the cytoplasmic surfactant organelles in AECII cells. The substitution resides in the LAMP domain adjacent to a conserved disulfide bond. In summary, this study describes a novel interstitial lung disease in dogs, identifies a new candidate gene for human surfactant dysfunction and brings important insights into the essential role of LAMP3 in the process of the LB formation.

## Author summary

We have characterized a lethal lung disease in neonatal Airedale Terrier dogs. The pathological features of the disease resemble those of the surfactant dysfunction in newborn babies. Surfactant is essential for lung function and we observed a maturation defect in the surfactant producing organelles of the lung epithelial type II cells. Genetic analyses revealed a recessive variant in the lysosome associated membrane *LAMP3* gene. LAMP3 is a structural, limiting membrane protein of the surfactant organelles. This study provides an excellent candidate gene for human surfactant disorders as well as new insights into LAMP3 biology and pathophysiology while the affected breed will benefit from genetic testing to eradicate this severe disease.

## Introduction

Pulmonary surfactant is a mixture of lipids and proteins essential for life that form a thin surface lining film in the gas exchange compartment of the lungs, the alveolus. Surfactant reduces the surface tension at the interface of air and liquid, preventing the alveoli from collapsing at the end of expiration. Surfactant is composed of lipids (~ 90%) with a small fraction (~10%) of mainly four surfactant proteins (SPs), SP-A, SP-B, SP-C and SP-D. The synthesis and assembly of surfactant occurs via distinct pathways within the AECII cells [1]. This processing requires developmental stages of special cytoplasmic organelles: first, multivesicular bodies (MVBs); second, composite bodies (CBs) and finally, mature LBs [2]. LBs store and secrete the produced surfactant into alveolar space by exocytosis [3]. AECII cell LBs are lysosome-related organelles that have common features with lysosomes, such as biogenesis, low internal pH and similarities in membrane components, yet they are cell-specific in morphology, function and composition [4,5].

Even a small defect that affects the complex pathway of LB formation or surfactant metabolism can have fatal consequences. Genetic dysfunctions of surfactant biogenesis that present in newborn infants constitute a subgroup of rare, diffuse childhood interstitial lung diseases that manifest as hypoxic respiratory distress or failure [6]. Three genes have been associated with primary congenital surfactant dysfunction in newborn babies: ATP-binding cassette, subfamily A, member 3, *ABCA3* (OMIM *601615, #610921); surfactant, pulmonary-associated protein B, *SFTPB* (OMIM *178640, #265120); and surfactant, pulmonary-associated protein C, *SFTPC* (OMIM *178620, #610913). The characteristic light microscopical changes include varying amount of proteinaceous material and macrophages in air spaces, AECII cell hyperplasia, arrested acinar development, lower number of alveoli and diffuse parenchymal changes of interstitial thickening with mesenchymal cells [7–9]. Overlapping morphological diagnoses can be made depending on the gene, duration of the condition and time point of biopsy [6].

Dogs have become excellent natural models for human disease as inherited disorders have been enriched in many dog breeds due to strict selective breeding schemes and breed structures [10,11]. With the rapid advancement of gene technologies, studies in dogs have unraveled novel disease genes and mechanisms [12–16]. These discoveries can advance the understanding of the corresponding human diseases, while animals and breeding programs will benefit from genetic testing.

Surfactant dysfunctions have not been previously reported as a spontaneous disease in neonatal animals. We describe here the first such example by identifying the genetic cause of a lethal diffuse interstitial lung disease in AT dogs with a novel missense variant in lysosome associated membrane 3, *LAMP3*. LAMP3 (also known as DC-LAMP, CD208) [17,18] localizes at the limiting membrane of surfactant organelles in AECII cells [19] and we demonstrate here how its defect arrests the maturation of LBs, resulting in a lethal deficiency of pulmonary surfactant in the affected AT puppies.

## Results

### Respiratory symptoms and lung-specific pathology in the affected puppies

All 25 affected puppies were born at term with normal delivery. One was stillborn. Six (24%) were lethargic at birth, refused to suckle and developed dyspnea or tachypnea and died or were humanely euthanized at 4–18 hours later. The rest of the puppies (72%) were initially normal until the difficulties in breathing started. Most of these puppies died during one to four days (64%), except one puppy that survived for 7 days and one up to 4 weeks.

**Fig 1:**
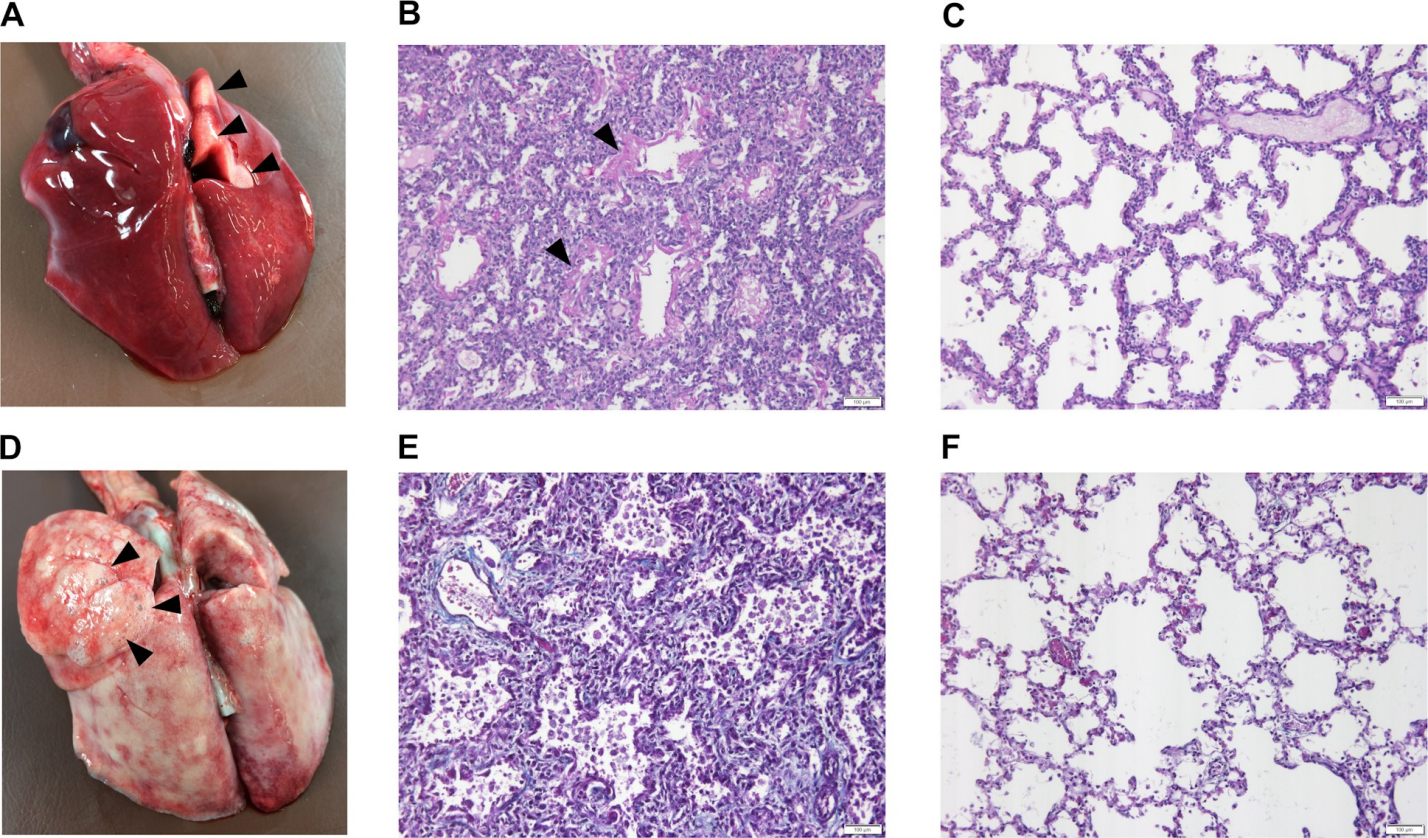
Macroscopical changes and histology of the affected and control lungs. (A) The lungs of the 3-day-old affected puppy are diffusely edematous and congested with only small visibly aerated area in the dorsal part of the right cranial lung lobe (black arrowheads). (B) In the affected lung of the 3-day-old puppy, the alveoli are small and collapsed in many areas and the alveolar walls are wide. The airspaces contain abundant eosinophilic material (black arrowheads) (Periodic acid Schiff (PAS) stain, 20X, scale bar 100 μm). (C) The morphology of the control lung corresponds to the normal saccular stage of development of 3-day-old puppies (PAS stain, 20X, scale bar 100 μm). (D) In the 28-day-old affected puppy, the lungs are rubbery in texture with multifocal emphysema (black arrowheads). (E) In the affected lung of the 28-day-old puppy, the alveolar septa are wide and contain increased amount of collagenous connective tissue (blue) and there are numerous macrophages and desquamated cells within the alveoli (Masson trichrome stain, 20X, scale bar 100 μm). (F) The morphology of the control lung of a 28-day-old puppy is normal for the alveolar stage of lung development. The alveolar septa are thin with normal amount of collagenous connective tissue (blue) (Masson trichrome stain, 20X, scale bar 100 μm).

Full necropsy was performed for the 25 puppies and the main lesion was in the lungs. The lungs of all the puppies, except the 4-week-old, were edematous, congested and appeared poorly aerated (Fig 1A). At four weeks the lungs had a rubbery texture and marked emphysema (Fig 1B). No significant macroscopic changes were detected in other organs.

The lungs of the stillborn puppy were at the normal saccular stage of maturation [20]. Some of the alveolar spaces contained strands and globules of Periodic acid Schiff (PAS) -positive eosinophilic material and macrophages. There was no atelectasis. In the puppies that died 4–18 hours after birth, the alveolar septa were wider than normal and there was copious amount of dense PAS-positive material in the airspaces and proximal bronchioles. There was no atelectasis but numerous macrophages in the airspaces. In the one to four days age group, there was multifocal atelectasis and emphysema in addition to changes described previously (Fig 1C and 1D). In the one- and four-week-old puppies, the emphysema was severe with intervening areas of atelectasia. There were numerous desquamated cells and macrophages in the alveoli and multifocal AECII cell hyperplasia. At four weeks, the lungs had a honeycomb pattern with rounded alveoli surrounded by thick interstitium containing increased amount of connective tissue indicating that the alveolar proteinosis had progressed to fibrosing interstitial lung disease (Fig 1E and 1F).

### Electron microscopy reveals a defect in the maturation and biogenesis of LBs

**Fig 2:**
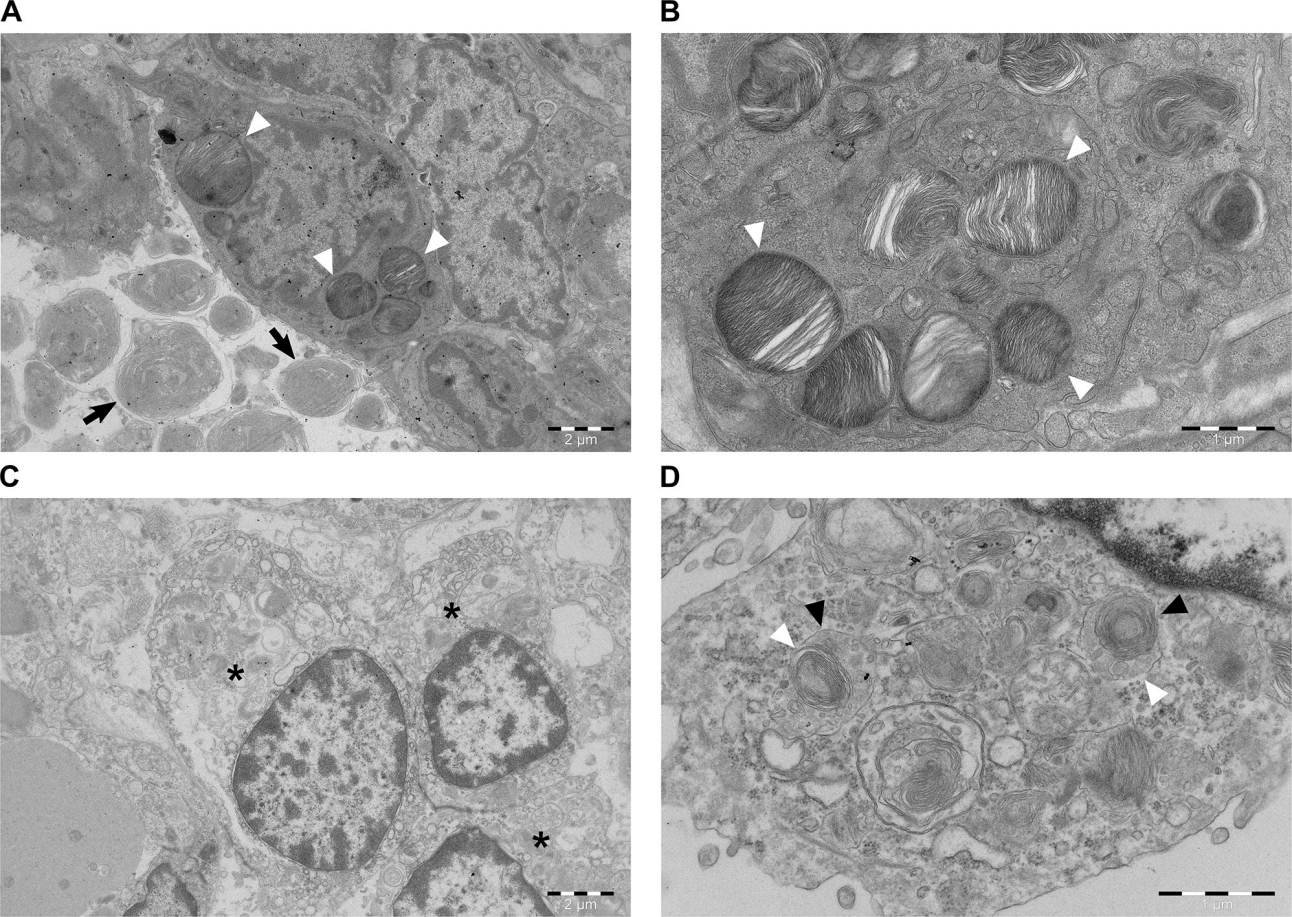
Transmission electron microscopy of the control and affected lungs. (A) Age-matched control. An intact AECII cell showing normal LBs with tightly packed lamellae (arrowheads). In the alveolar airspace, surfactant is still in the form of multiple concentric lamellar whorls (arrows) (scale bar 2 μm). (B) Age-matched control. Multiple cytoplasmic LBs showing intact limiting membranes with intraorganellar, parallel packed phospholipid lamellae (arrowheads) (scale bar 1 μm). (C) Affected puppy. AECII cell hyperplasia in the alveolar wall with three severely altered AECII cells (asterisks) in apposition (scale bar 2 μm). (D) Affected puppy. A high power view of the cytoplasm of an AECII cell showing structures compatible with CBs (black arrowheads) without any mature LBs. The limiting membrane is occasionally disrupted (white arrowheads) (scale bar 1 μm).

For the characterization of ultrastructural changes in the lung, we performed transmission electron microscopy (TEM) in one affected and one age-matched, unaffected control puppy. In the control, the preservation and ultrastructural appearance of the alveolar epithelium was excellent with AECII cells present as single cuboidal cells next to thin cell extensions of type I alveolar epithelial cells (AECI) (Fig 2A). The characteristic surfactant-containing secretory organelles in AECII cells were mainly visible as mature LBs with tightly packed lamellae surrounded by a limiting membrane (Fig 2B).

In the affected animal, the ultrastructure of AECII cells was severely altered. The AECII cells appeared in clusters, which indicates cell hyperplasia. Their nuclear chromatin was marginalized, the perinuclear cisterna was dilated, the mitochondria were swollen, and the cytoplasmic matrix was less dense (i.e. less electron scattering) (Fig 2C). The secretory organelles were smaller and contained fewer lamellae, often in combination with small vesicles surrounded by an occasionally disrupted common limiting membrane (Fig 2D). These organelles were immature and represented CBs, the intermediate step in LB biogenesis between MVBs and mature LBs.

### Genetic analyses identify a variant in *LAMP3*

**Fig 3:**
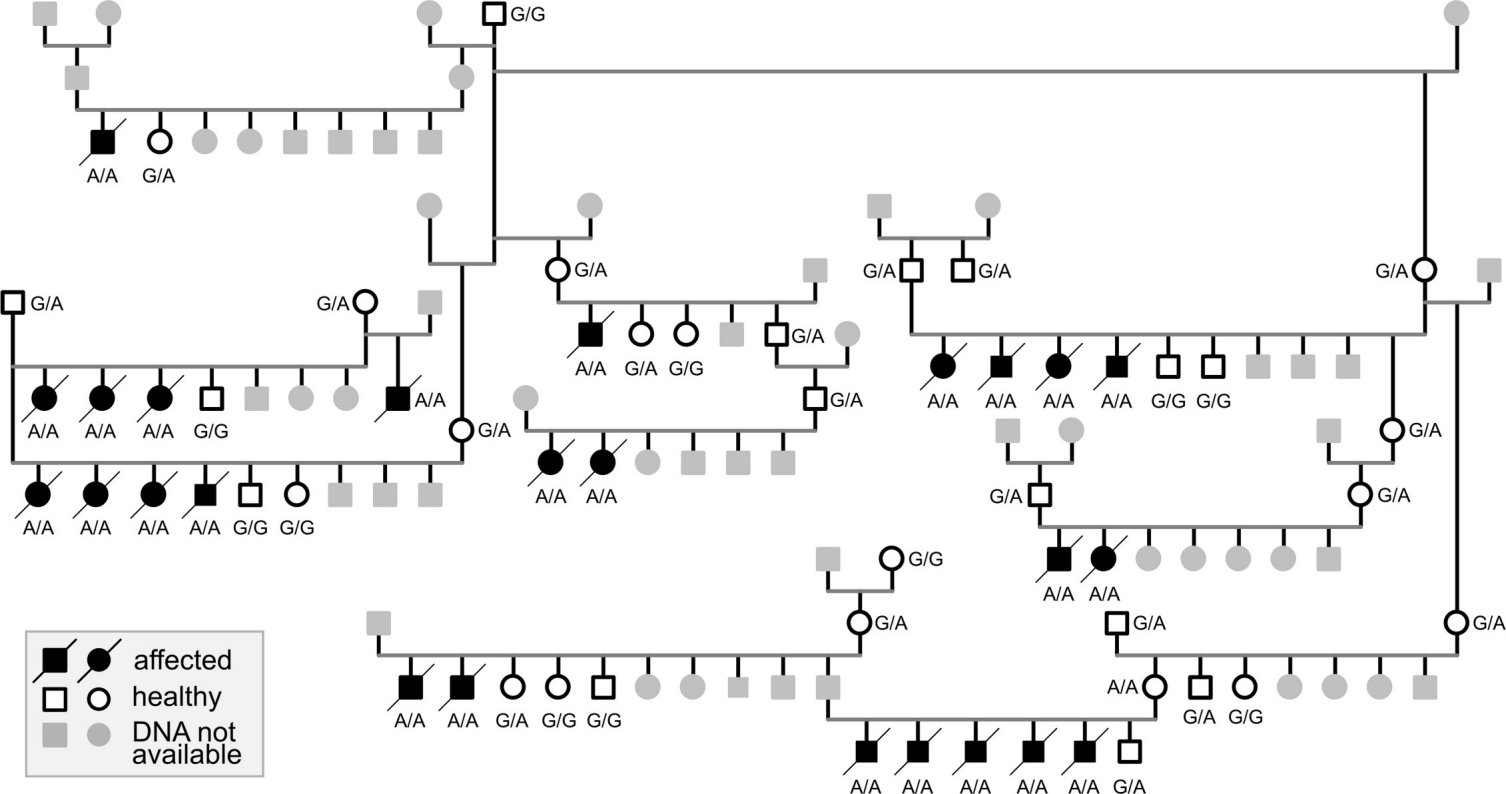
A pedigree of the 25 affected puppies. The segregation of the *LAMP3* variant in the pedigree indicates autosomal recessive inheritance. Black symbol denotes homozygous mutant (A/A), half-filled denotes heterozygous carrier (G/A) and white symbol denotes wild type (G/G). The individuals with gray symbol were not available for genotyping.

**Fig 4:**
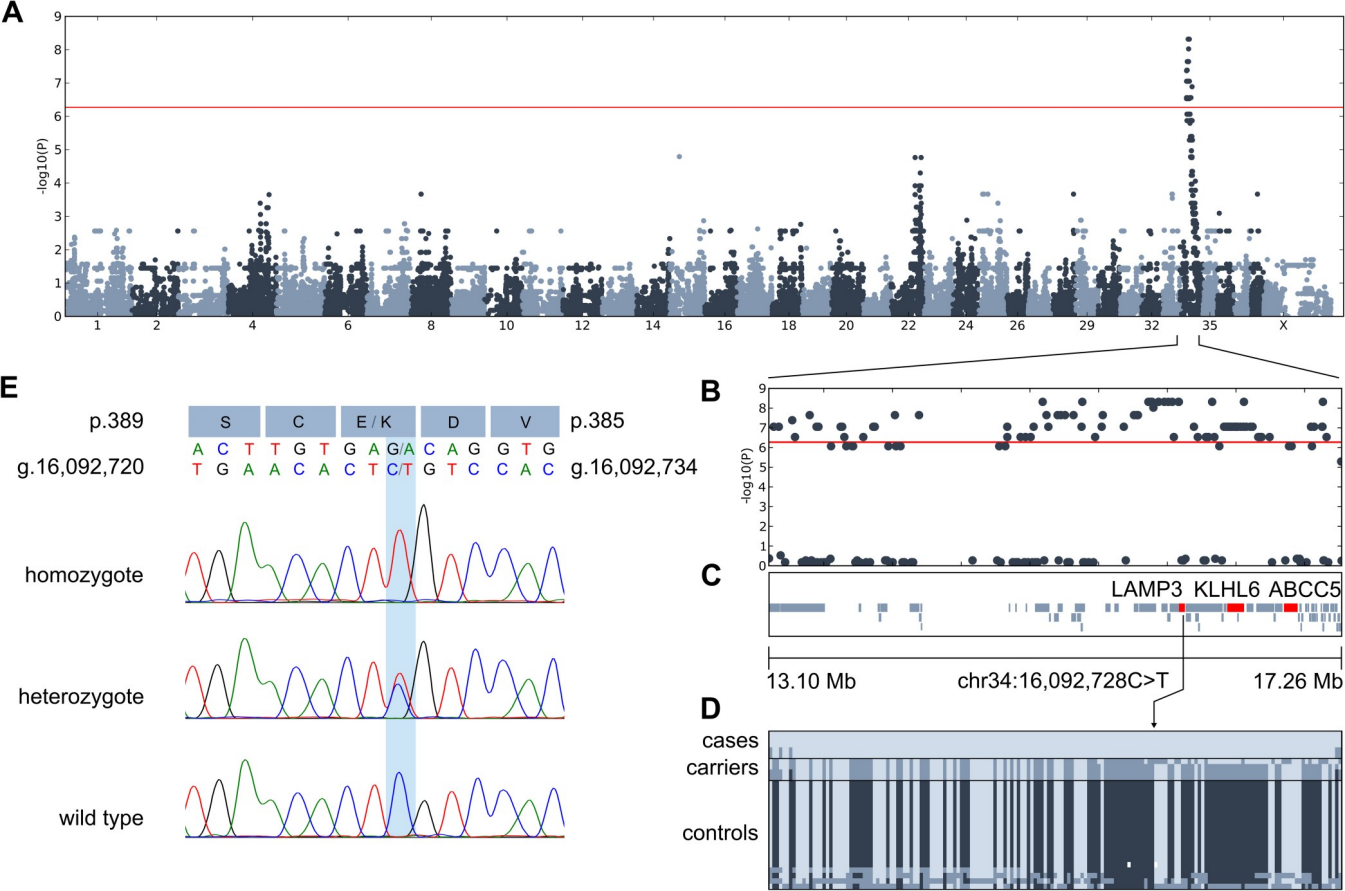
Genetic analyses reveal a missense variant in *LAMP3*. (A) The results of a GWAS with 5 cases and 24 controls reveal an association in chromosome 34. The most significant markers achieve genome-wide significance (p_raw_ = 7.161×10^−10^, p_Bonf_ = 6.689×10^−5^). The genome-wide significance threshold at 5.5×10^−7^ is indicated in red. (B) A regional association plot of the locus in chromosome 34. (C) The associated 3.9 Mb region in chromosome 34 contains 57 genes as annotated by NCBI (release 105), including *LAMP3*, *KLHL6* and *ABCC5*. (D) The genotype data shows a shared homozygous 3.9 Mb haplotype block in the affected puppies. The location of the *LAMP3* variant is denoted with an arrow, as the distances between markers in the genotype image do not directly correspond to images C and D. (E) Chromatograms and the nucleotide and consequent amino acid change (p.(E387K)) of the *LAMP3* missense variant. The *LAMP3* gene is coded on the reverse strand. The chromatograms are shown for homozygous mutant (A/A), heterozygous (G/A) and wild-type (G/G) individuals.

As the pedigree of the affected puppies was suggestive of an autosomal recessive inheritance for the disease (Fig 3), we combined genome-wide association study (GWAS) and a whole-exome sequencing (WES) approaches to find the candidate causative variant. A GWAS with 5 affected and 24 unaffected AT dogs revealed 198 SNPs with genome-wide significance on chromosome 34 between 12,013,938–22,402,691 bp (S1 Table). Fifteen most significant SNPs (p_raw_ = 7.161 × 10^−10^, p_Bonferroni_ = 6.689 × 10^−5^) were located at 15,092,907–17,132,621. Assessment of the genotypes revealed a 3.9 Mb homozygous region at 13,290,333–17,170,957 in the affected puppies (Fig 4A–4D). The analysis of WES data from two affected and one obligate carrier yielded 347 case-specific homozygous variants, of which 22 were exonic. Only three private coding variants in *LAMP3*, *ABCC5* and *KLHL6* resided in the associated region in the affected puppies (Table 1) and were not observed in 197 unaffected dogs from 67 breeds and 3 wolves (S3 Table). *LAMP3* is properly annotated in NCBI (release 105, Gene ID: 607186) but, to our understanding, erroneously interpreted as MCF2L2-201 transcript (ENSCAFT00000018703.3) in Ensembl (release 95) and UCSC. The genotyping of the three variants in 16 affected puppies and 15 close relatives revealed that all the affected dogs were homozygous for the alternate alleles whereas unaffected obligate carriers were heterozygotes and controls wild type, suggesting a strong linkage disequilibrium between the variants.

**Table 1:** Case-specific coding variants found in whole exome sequencing data in the associated locus.

Gene	Protein	Genomic coordinates	Coding sequence variant	Amino acid consequence	Predicted effect
*LAMP3*	lysosomal-associated membrane protein 3	chr34:16092728C>T	c.1159G>A XM_843796.4	p.(E387K) XP_848889.2	Deleterious
*ABCC5*	ATP-binding cassette subfamily A member 5	chr34:16887330G>A	c.2524C>T NM_001128100.1	p.(P842S) NP_001121572.1	Neutral
*KLHL6*	kelch-like family member 6	chr34:16447091G>A	c.1713C>T XM_545220.6	p.(I571I) XP_545220.3	No effect

**Fig 5:**
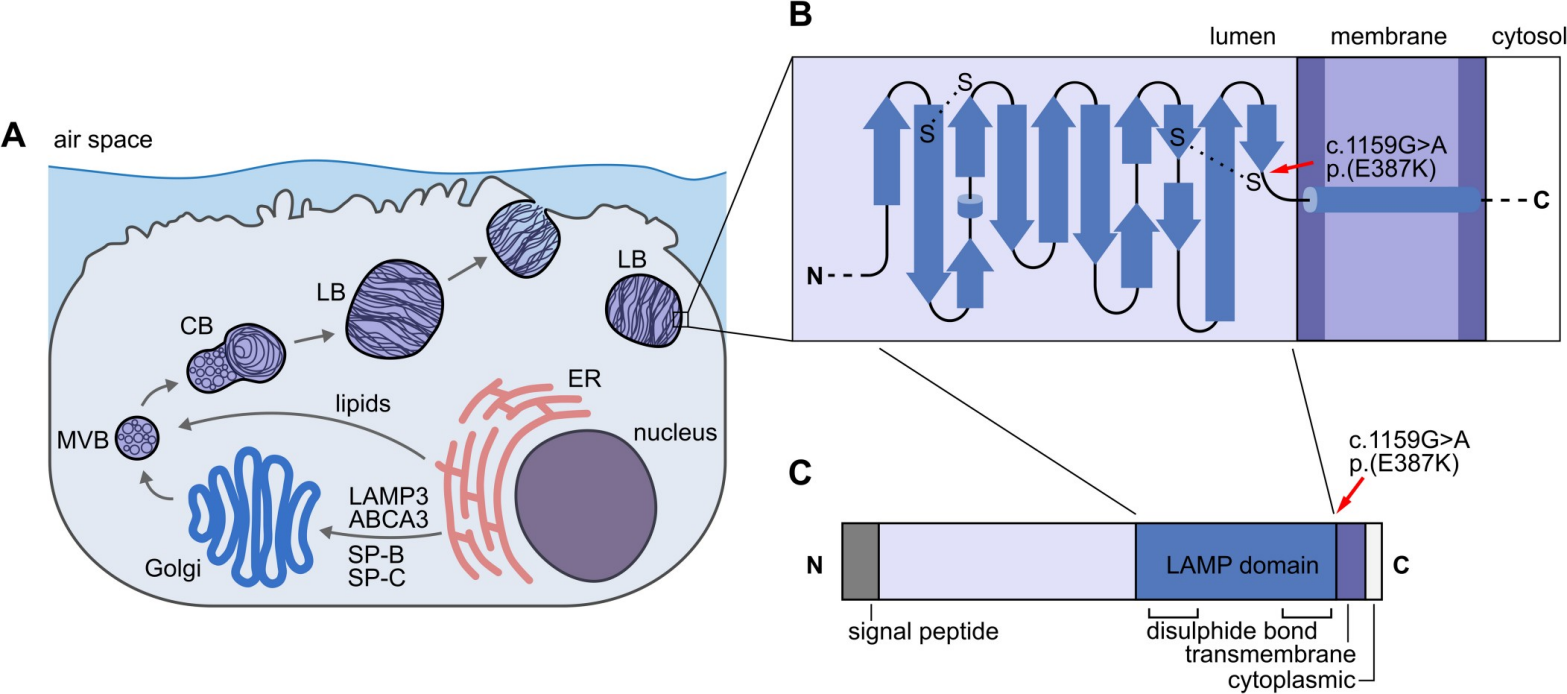
The LAMP3 variant in the affected puppies. (A) A schematic representation of a lung AECII cell. During surfactant production, ABCA3, SP-B, SP-C and LAMP3 are delivered to the LBs via endoplasmic reticulum (ER), Golgi, MVBs and CBs whereas lipids pass Golgi on their path to LBs. (B) A simplified topology diagram of the LAMP domain in the LAMP3 protein. The p.(E387K) variant (denoted by red arrow) resides next to a conserved cysteine residue that participates in the formation of the second disulfide bond. (C) A schematic representation of all the domains in the LAMP3 protein.

KLHL6 is involved in B-lymphocyte antigen receptor signaling and its variants have been associated with B-cell lymphomas and chronic lymphocytic leukemias [21]. Given the gene’s unlikely role in primary lung disease and that the identified *KLHL6* c.1713C>T, p.(I571I) variant was synonymous, we focused further analyses on the nonsynonymous variants in *LAMP3* and *ABCC5*. ABCC5 is ubiquitously expressed in tissues and acts as a general transporter of glutamate conjugates [22] and Abcc5^-/-^ mice have a normal cellular phenotype [23]. LAMP3 is a lysosome-associated membrane protein that is co-expressed in the limiting membrane of alveolar AECII cell LBs with ABCA3, whose defects can result in surfactant dysfunction (Fig 5A) [19,24]. Altogether, we genotyped the *LAMP3* and *ABCC5* variants in 371 affected and control AT dogs and found that both variants fully segregated with the disease under recessive model (25/371 homozygous mutant, 77/371 heterozygous carrier, 269/371 homozygous wild type) (Fig 4E). In this cohort, the carrier frequency was 20.6%.

### Whole genome sequencing

To further investigate the structural and non-coding variant content in the associated locus, we performed whole genome sequencing in one affected dog. The WGS data from this dog was filtered against 887 control genomes (S4 Table) and revealed only two private coding variants (Table 2, S5 Table). The two coding variants in *LAMP3* and *ABCC5* were the same as previously discovered by WES (Table 1). The WGS analysis revealed many case-specific intronic or intergenic variants but they were excluded as candidates because they were not located in highly conserved regions (S5 Table). Structural variant and mobile element analysis revealed 548 structural variants and 446 mobile elements in the associated region. Only one predicted case-specific intergenic SINE was observed in the locus (chr34:14462208) but was not considered as a candidate for the disease as the variant was intergenic.

**Table 2:** Variants identified in the whole genome of the affected dog.

total number of variants	6740456
variants in the locus region (chr34: 13,290,333–17,170,957)	79066
of which homozygous	78811
of which private	24
of which exonic	2

### In silico evaluation of the pathogenicity of the candidate variants

The pathogenicity of the two candidate variants from WES and WGS analyses was assessed with Provean and PredictSNP [25,26]. The ABCC5 p.(P842S) variant was predicted as neutral (Provean score -0.005, cutoff -2.5 and PredictSNP 83% confidence) and the LAMP3 p.(E387K) variant as deleterious (Provean score -3.430, cutoff -2.5 and PredictSNP 72% confidence). The LAMP3 p.(E387K) amino acid change resides in the LAMP domain just adjacent to the fourth conserved cysteine residue that participates in the second disulfide bond (Fig 5B and 5C). Furthermore, the conservation of p.E387 was evaluated across 36 species with multiple alignment generated with Clustal Omega [27]. The result suggests conservation of the residue (S6 Table). Collectively, these results highlight *LAMP3* and its predicted deleterious variant as a likely cause of the recessive disease in the AT breed.

### Screening of the *LAMP3* variant

After the initial cohort of 371 AT dogs that were screened for the *LAMP3* variant, owners had submitted blood samples from 20 additional adult, non-affected AT dogs. As they were screened, one of these dogs, a dam that had had a litter that included affected puppies, turned out to be homozygous for the *LAMP3* variant (Fig 3). At the time of sampling, the dam was 6 years of age and, according to its owner, had been clinically healthy all its life without any signs of respiratory disease. To study the possible differences of the variants in the associated locus between the dam and the affected puppy, the whole genome of the dam was also sequenced and compared with the affected puppy. This analysis did not reveal any dam-specific heterozygous variants, which would have indicated that our *LAMP3* variant is not causal but instead in LD with the true disease-causing variant. In contrast, this result suggests that the dam may have an unknown protective variant. Recently, modifier variants that afford resilience to severe disease-causing variants have been found, suggesting that incomplete penetrance in Mendelian diseases is not as rare as previously thought [28–31]. Finally, to investigate the breed specificity of the *LAMP3* variant, we screened additional 6940 dogs from 297 breeds, including eight Airedale Terriers (S7 Table). Only one heterozygous dog, an Airedale Terrier, was identified, which indicates that the *LAMP3* variant is specific to the breed.

### Immunohistochemistry of LAMP3, SP-B and SP-C in the lung

**Fig 6:**
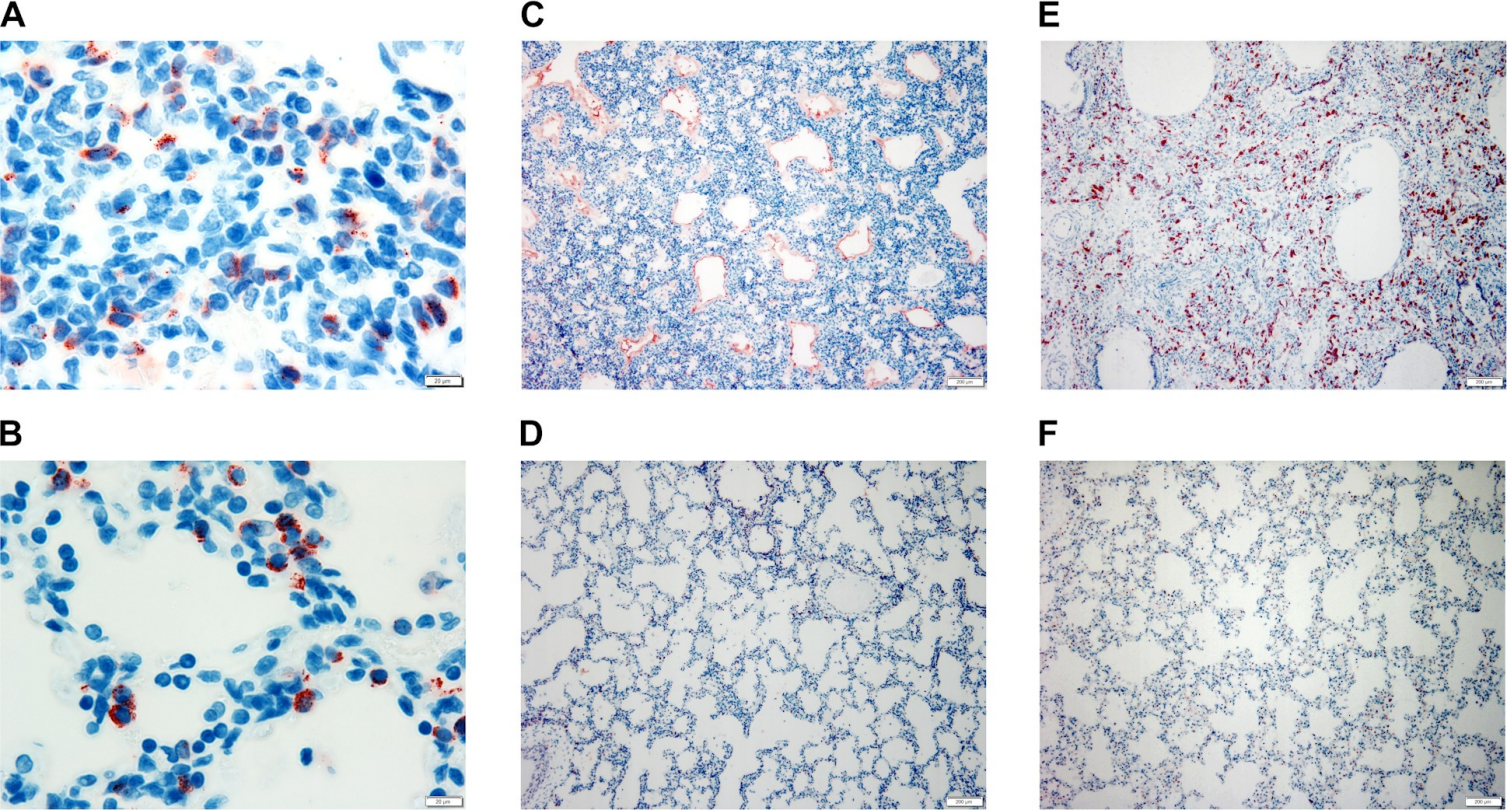
LAMP3, SP-B and SP-C immunohistochemistry. In the affected (A) and age matched 3-day-old control puppies (B), LAMP3 protein expression was detected as granular, globular to ring-like intracytoplasmic structures (red) in the AECII cells (100X, Z-stack, scale bar 20 μm). In the affected 3-day-old puppy (C), the proteinaceous material in the airspaces stained intensely with SP-B antibody (red), whereas in the age-matched control (D), SP-B positive staining was detected only in the cytoplasm of AECII cells and the epithelium of few bronchioles (10X, scale bar 200 μm). In the 28-day-old affected puppy (E), the significant increase of the SP-C positive cells (red) indicated AECII hyperplasia. In comparison, the age-matched control (F) had only scattered SP-C positive AECII cells in the alveolar epithelium (10X, scale bar 200 μm).

It is possible that the LAMP3 p.(E387K) amino acid substitution may distort the native conformation of the protein or affect the expression, intracellular processing or localization of LAMP3. Furthermore, as a limiting membrane protein, it may also have an effect on the processing of surfactant proteins. To evaluate the effect of the variant, we assessed the expression of LAMP3, SP-B and SP-C by immunohistochemistry. In the affected and control puppies, there were similar LAMP3 positive cytoplasmic granular to small globular and ring like structures in the cytoplasm of the alveolar epithelial cells (designated as AECII cells). Moreover, there was no difference in the intensity of staining between the groups (Fig 6A and 6B). In the one- and four-week-old puppies, there was slightly higher number of positive cells and many positively stained desquamated intra-alveolar cells and possibly macrophages indicating phagocytosis of the desquamated AECII cells. Hence, the mutant LAMP3 protein is equally expressed and localized in the alveolar AECII cells compared to native LAMP3.

The stillborn puppy had occasional SP-B positive strands in the alveolar spaces in addition to normal positive staining of a few alveolar lining cells (designated as AECII) and numerous bronchiolar epithelial cells. In the affected dogs, the bronchiolar staining decreased and alveolar staining increased with age and in the one- and four-week-old puppies the number of positive alveolar epithelial cells was significantly higher in the affected puppies compared to age-matched controls indicating AECII hyperplasia. The alveolar PAS positive material stained intensely positive with SP-B (Fig 6C and 6D). In the one- and four-week-old puppies, there was also positive staining of the desquamated cells within the alveoli.

With the polyclonal SP-C antibody, there was positive cytoplasmic staining of the alveolar epithelial cells (designated as AECII). In the affected puppies, the number of positive cells increased with age and in the one- and four-week old puppies the number was significantly higher in the affected puppies compared to age-matched controls, indicating hyperplasia (Fig 6E and 6F).

## Discussion

This study provides new insights to the biology of surfactant dysfunctions with a novel disease gene, *LAMP3*. First, we describe a spontaneous interstitial lung disease in dogs with clinical symptoms and pathology corresponding to the most severe congenital forms of human surfactant dysfunctions associated with abnormal LB formation in the alveolar AECII cells. Second, we found that LBs were underdeveloped and in the developmental stage of CBs in the affected AECII cells, which demonstrates an impairment in the formation of mature LBs. Furthermore, the common limiting membrane of CBs was occasionally disrupted, suggesting a defect in the formation of the limiting membrane. Finally, we identify a variant in *LAMP3*, which is a highly relevant candidate gene in these disorders.

The pathology of the affected puppies corresponds to the severe disorders of surfactant biogenesis in human infants. The typical histological changes of proteinaceous material and consequential macrophage accumulation in the air spaces as well as AECII hyperplasia can be attributed to defective surfactant production and subsequent damage to the alveolar epithelium. AECII hyperplasia occurs as a well-known reparative response to a damaging insult to the alveolar epithelium with the eventual transformation of AECII into AECI cells in this process [32,33]. The pathogenesis of the characteristic arrest of the acinar development and interstitial thickening with mesenchymal cells has not been closely studied. In the stillborn affected puppy, the acinar structure was in the saccular stage of development, which is normal for at term puppies [20] and interstitial thickening was not apparent at this stage, whereas the aforementioned abnormalities were clearly seen in the older affected puppies. This suggests that the acinar and interstitial changes manifest and progress during breathing and that these changes could be secondary and reflect a reparative mechanism to the injured alveolar septa and atelectasis. Alternatively, they could be a consequence of a yet unknown process.

In the TEM analysis of the affected lungs, AECII hyperplasia was evident in the alveolar epithelium. Moreover, the observed ultrastructural damage to the epithelium and underlying basal lamina had preceded the intra-alveolar accumulation of proteinaceous oedema fluid. This was seen as floccular material mixed with unilamellar vesicles of surfactant in the alveolar space in contrast to the normal lamellar body-like to tubular myelin structures of surfactant in the control lung [34]. The severe ultrastructural alterations of the intracellular organelles in the affected AECII cells were typical for ischemic change that can occur secondary to alterations in surfactant composition or function, whether the primary mechanism is genetic or ischemic or due to reperfusion injury in e.g. lung transplantation [2,35]. In the affected but still intact AECII cells, LBs were underdeveloped, in the developmental stage of CBs with occasional disruption of the limiting membrane, which clearly indicates an impairment in the formation of this membrane and subsequent defect in LB formation. The arrest in maturation cannot be attributed to ischemia, since in an experimental study on ischemia related ultrastructural changes in canine AECII cells, the cytoplasmic surfactant organelles were well preserved [35]. A delayed post-mortem period before fixation can certainly cause similar changes as in ischemia, but this post-mortem artefact is unlikely as the sampling of both the affected and control puppy had occurred at a similar post-mortem interval, and the ultrastructure of the control lung was well preserved. Without the proper amount and composition of surfactant, the alveoli collapse as the surface tension increases and a progressive vicious cycle develops as the areas of atelectatic alveoli are no longer ventilated and perfused due to hypoxic vasoconstriction [35].

In the affected AT puppies, the observation of improperly matured LBs in the affected AECII cells supports our hypothesis of a primary defect in the surfactant metabolism as the underlying mechanism for the disease. As the human surfactant deficiencies caused by pathogenic variants in *ABCA3*, *SFTPB* and *SFTPC* have distinct ultrastructural changes in the LBs, TEM has been the golden standard for diagnosis prior to the genomic methods available today. The ultrastructural feature of arrested LB maturation that is observed as structures compatible with CBs in the cytoplasm without normally formed, mature LBs in the affected puppies is also the hallmark finding in human SP-B deficiencies [36,37]. In contrast, the typical findings of human ABCA3 and SP-C deficiencies are different to what we observed in the affected puppies. As for ABCA3 deficiency, depending on the pathogenicity of *ABCA3* variants, the LBs have a distinct ultrastructural morphology of "fried eggs” as the abnormal LBs have tightly packed lamellae and an aggregation of electron dense material [7,36]. In human SP-C deficiencies, the intracytoplasmic LBs appear to be enlarged and contain disorganized lamellar structures [38], which is in contrast to SP-C deficient mice that have no observable ultrastructural changes in the LBs [39].

LAMP3 was originally found to be transiently expressed in the MHC class II compartment of dendritic cells [17]. Liao et al. [40] showed that LAMP3 regulates hepatic lipid metabolism, which suggests that LAMP3 can be of relevance as a drug target in disorders of lipid metabolism. In cancer biology, e.g. overexpression of LAMP3 is associated with potential metastatic cervical cancer [41]. As LAMP3 is expressed in the limiting membrane of LBs in alveolar AECII cells, it is an excellent candidate for surfactant dysfunction [19].

LAMP3 belongs to the lysosome-associated membrane protein family that share a conserved LAMP domain [42]. LAMP3 is a highly glycosylated single transmembrane protein with an intraorganellar N-terminal signal peptide, a LAMP domain with two conserved disulfide bonds, multiple O- and N-linked glycosylation sites and a transmembrane helix before a short cytoplasmic C-terminal tail [42]. Wilke et al. [42] revealed the secondary crystal structure of the human LAMP domain in LAMP3. This domain has a unique β prism fold that is stabilized by two disulfide bonds at the cysteine residues. The prism structure is conserved across LAMP domains and is formed by two β-pleated sheets that are bent by β-bulges and connected by the second disulfide bond [42,43]. The LAMP3 p.(E387K) variant resides within the LAMP domain adjacent to the fourth cysteine residue that is involved in the formation of the second disulfide bond, which stabilizes the C-terminus of the protein in the center of the β-prism [42]. Multiple alignment of LAMP3 amino acid sequences across species indicates that p.E387 is conserved (S6 Table). Although we lack functional evidence at this stage, it is possible that the change from negatively charged glutamic acid into positively charged lysine next to the S-S bond may distort the native conformation of the LAMP domain and thus disturb the interaction with other limiting membrane proteins in the process of mature LB formation. As LB maturation in the affected puppies had ceased at the CB stage, a failure in the formation of a proper limiting membrane is plausible. Without a proper limiting membrane, the acidic intraorganellar environment is disturbed, which consequently affects the posttranslational proteolytic processing of the limiting membrane glycoprotein ABCA3 and surfactant proteins SP-B and SP-C, as the necessary enzymes are not properly enclosed within the MVBs, CBs and LBs [44–46]. Moreover, without the normal function of ABCA3 as a transmembrane lipid transporter in the limiting membrane, further detrimental effect in surfactant biogenesis ensues, as the transportation of lipids into the surfactant processing organelles ceases [47,48]. This aforementioned combined global dysfunction most likely explains the severity of the disease in the affected puppies.

We were unable to study the functional consequence of the *LAMP3* variant at protein level since neither snap frozen tissue samples nor bronchoalveolar lavage fluid were available from the affected puppies. However, LAMP3 IHC on paraffin embedded lung tissue indicated similar expression of the protein in the affected and control lungs, suggesting a pathogenic conformational defect in the mutated protein. In the affected one to four days old puppies, the intra-alveolar proteinaceous material stained intensely positive with SP-B antibody, whereas it stained only slightly with SP-C. This suggests a possible overproduction of SP-B and a reduction of SP-C in the affected puppies, since a similar relative change in the amount of these proteins has been reported in a patient with a homozygous missense *ABCA3* variant [49]. This abnormality in SP-B and SP-C production in the affected puppies and child with *ABCA3* variant might be caused by a comparable underlying mechanism.

*LAMP3* is a novel candidate gene for human surfactant deficiencies. A syndrome caused by 3q26.33-3q27.2 microdeletion haploinsufficiency overlapping *LAMP3* has been recently proposed in children [50,51]. In these cases, possible pathological changes in the lungs were not confirmed by biopsy, thus making the pathogenicity of *LAMP3*^+/-^ uncertain. However, the children responded to therapy and clinical symptoms were mild compared to the affected puppies, suggesting that the haploinsufficiency with 3q26.33-3q27.2 microdeletion syndrome yields enough functional protein to maintain pulmonary function to avoid lethality. By sequencing the known disease-causing variants or the whole candidate gene in surfactant disorders, the underlying genetic defect is commonly found. However, Somaschini et al. [52] have reported lethal respiratory distress in three newborn infants without pathogenic coding or intron-exon boundary variants in *ABCA3*, *SFTPB* or *SFTPC* and they speculated of a yet unknown underlying genetic mechanism. Our study suggests that in unresolved cases of interstitial lung disease in neonatal babies, variants in *LAMP3* should be closer investigated.

In conclusion, we have characterized a neonatal interstitial lung disease in a dog breed with a missense variant in AECII cell LB limiting membrane protein LAMP3. This finding provides a novel candidate gene and new insights to LB biology. Furthermore, a genetic test can be developed for veterinary diagnostic and breeding purposes to eradicate this severe disease from the AT breed.

## Materials and methods

### Ethics statement

The tissue samples were collected during necropsy and the blood sample collection was ethically approved by the Animal Ethics Committee of State Provincial Office of Southern Finland (ESAVI/7482/04.10.07/2015). All dogs had been voluntarily sent for necropsy by the breeder or owner.

### Study animals and pedigrees

The study cohort comprised of 371 AT dogs and included altogether 44 puppies and 3 adult dogs that had been sent for a pathological examination. Unaffected age-matched puppies from other breeds were used as controls in this study. EDTA blood samples were collected from 324 unaffected AT dogs, including parents, siblings and close relatives of the affected puppies. The pedigree of the affected puppies was visualized with the GenoPro software (version 2.5.4.1). Pedigree information was obtained from the public database of the Finnish Kennel Club [53].

### Postmortem examination

A necropsy was performed on all affected puppies. Samples for histology from all lung lobes (multiple sections), major organs and macroscopically abnormal tissues were collected and fixed in 10% buffered formalin, routinely processed and embedded in paraffin. Lung samples were cut into 2 μm, other organs into 4 μm sections and all were stained with Hematoxylin and Eosin (HE) and examined by light microscopy. Special stains Periodic Acid-Schiff (PAS) for glycoprotein and Masson Trichrome (MTRI) for fibrosis were used on lung tissue (4 μm sections). Lung tissue samples collected during necropsy from age-matched unaffected AT puppies and puppies from other breeds were used as controls.

### Transmission Electron Microscopy (TEM)

A 1.5-day-old affected puppy and an age-matched unaffected puppy from another breed had been dead for two hours and kept at 4°C prior to necropsy. Lung tissue samples were immediately immersed into 5% glutaraldehyde in 0,16 M s-collidin buffer, pH 7,4 during necropsy. The samples were stored in this media and sent to University of Turku, Laboratory of Electron Microscopy. Next, the samples were washed in s-collidin buffer three times, 3 min each. Post fixation was done with 2% OsO4 + 3% K-ferrocyanide (1:1) for 2 hr [54]. After post fixation, the samples were washed three times in s-collidin buffer, 5 min each. The dehydration process was done using ascending concentration of ethanol starting with 70% ethanol, 1 min at +4°C; 96% ethanol, 1 min at +4°C; 100% ethanol, 30 min at +4°C; and finally, 100% ethanol, 3 times, 30 min each at +20°C. For embedding, the samples were first kept in propylene oxide two times, 15 min each; next, in propylene oxide with epoxy resin and DMP (10:10:0,15) for 2 hours prior to epoxy resin with DMP (10:0,15) for 12 hours; and finally, epoxy resin with DMP (10:0,15) in +60°C for 36 hours. The sections were cut with an ultramicrotome to a thickness of approximately 70 nm. The samples were manually stained first with 1% uranyl acetate in pure water for 30 min, after which they were rinsed in pure water three times, 30 sec each and the final staining was done with 0.3% lead citrate in pure water for 3 min, following three rinses in pure water, 30 sec each. The micrographs were taken using JEM-1400 Plus transmission electron microscope (JEOL, Japan) operated at 80 kV.

### Genotyping and GWAS

Illumina´s CanineHD Beadchip containing 173,662 SNPs was used for the genotyping of 5 affected puppies and 24 unaffected relatives at Geneseek (Neogen Corporation). A case-control association study (GWAS) was performed with PLINK version 1.07 software [55]. Parameters for the quality control were marker and sample call rate of > 95%, minor allele frequency (MAF) of > 1% and Hardy-Weinberg equilibrium of p > 0.0001. No individual dogs were removed after genotype pruning and frequency test; in total, 93401 SNPs remained in the analysis. Multiple testing correction was implemented with the Bonferroni method, and genome-wide significance level was subsequently set to 5.353 x 10^−7^. Population stratification was assessed with genomic inflation factor (lambda) and from a QQ plot after analysis (S1 Fig). The QQ plot indicated a slight stratification in the cohort, which is typical for association studies in highly inbred and structured populations.

### Whole exome sequencing

Two affected puppies and one obligate carrier of the AT breed were whole exome sequenced by using Roche NimbleGen SeqCap EZ target enrichment design 140702_CanFam3_exomeplus_BB_EZ_HX1 kit with a total capture size of ~152 Mb [56]. The sequencing was performed with Illumina NextSeq500 at the Biomedicum Functional Genomics Unit (FuGU, University of Helsinki). The acquired reads were mapped with the Burrows-Wheeler Aligner (BWA) 0.7.12-r1039 version [57] and the mapped reads were categorized and the duplicates marked with Picard tools [58]. Genome Analysis Tool Kit (GATK) HaplotypeCaller version 3.5.0 [59] was used for indel realignment, base-quality score realignment and variant calling.

For the identification of candidate variants, we performed filtering under an autosomal recessive model using Genotype Query Tools [60,61] and an in-house variant database. Before filtering, approximately 240 000 variants were present in each sample, out of which approximately 139 000 were homozygous (S2 Table). First, we selected variants that were homozygous in the two affected puppies and heterozygous in the obligate carrier. To further narrow down the set of candidate variants with control genomes, we utilized publicly available data from 197 unaffected dogs from 67 breeds and 3 wolves (S3 Table). CanFam3.1 was used as the reference genome. For *LAMP3*, we used nucleotide acid sequence XM_843796.4 and XP_848889.2 protein sequence. The whole exome sequences of three dogs have been deposited to the NCBI SRA database with the accession number PRJNA515838.

### Whole genome sequencing

One affected and one unaffected AT dog were whole genome sequenced by Illumina high-throughput sequencer with paired-end strategy at a read length of 300 bp (2 x 150bp). The affected and unaffected dog had an average coverage of 25X and 33X, respectively. The read processing was performed as described above. In addition, structural variants (SVs) including deletions, insertions, duplications, and intra-chromosomal re-arrangements were identified with DELLY [62]. The consensus sequence for LINEs of type L1-Y_Cf (young active repeats) and for the SINEC1_CF and SINEC2_CF families were obtained from Repbase database [63]. The Mobile Element Locator Tool (MELT) [64] was then used to discover and genotype the non-reference mobile element insertions (MEI), namely SINEs and LINEs, in the sequenced dogs. The SVs and MEIs were filtered assuming autosomal recessive mode of inheritance, where the variant was required to be homozygous in the affected dog and wild-type in 257 additional unaffected dogs.

### Immunohistochemistry (IHC)

Formalin fixed, paraffin embedded lung samples were cut into 2 μm sections. The following primary antibodies and dilutions were used: Anti-mouse DC-LAMP/CD208 rat monoclonal antibody (1:100, DDX0191, Dendritics) that has previously been used on dog tissue [65]. Anti-human SFTPB /Surfactant protein B rabbit polyclonal antibody (1:400, LS-B8081, LifeSpan BioSciences LSBio) that corresponds with 86% similarity to residues 154–202 of the canine surfactant protein B (XP_013975904.1), and SFTPC anti-human rabbit polyclonal antibody (1:200, PA5-76631, Thermo Fisher) that is produced against the recombinant full-length human protein that corresponds with 79% identity to dog surfactant protein C (XP_534578.4). The sections were deparaffinized in UltraClear (J.T Baker) and rehydrated in graded ethanol series. Heat induced antigen retrieval in citrate buffer (pH 6) for 20 minutes in microwave was performed for DC-LAMP/CD208 and SFTPB whereas no retrieval method was used for SFTPC. Fresh 1% of H_2_O_2_/MetOH for 10 minutes was used to block endogenous peroxidase activity in all samples prior to 5 minutes in Ultra Vision Protein Block (UltraVision LP Detection System HRP Polymer & AEC Chromogen, Thermo Scientific). Sections stained with DC-LAMP/CD208 and SFTPB antibodies were incubated overnight in +4°C and SFTPC sections in room temperature (RT) for 1 hour. BrightVision (ImmunoLogic) post-antibody blocking solution (for 15 minutes) was used for DC-LAMP/CD208 stained sections and UltraVision Primary Antibody Enhancer (20 minutes in RT) for the sections stained with the other two antibodies. The SFTPC stained sections were incubated for 30 minutes in RT whereas SFTPB sections were incubated for 45 minutes in +37°C with the secondary antibody, UltraVision HRP polymer. BrightVision Poly-HRP-Goat anti Mouse/Rabbit IgG secondary antibody (ImmunoLogic) was used for DC-LAMP/CD208 according to manufacturer’s instruction. UltraVision AEC Single Solution chromogen (Thermo Fisher) was used for all sections to visualize the reactions as well as counterstaining with Meyers hematoxylin prior to Aquatex (Merck) mounting.

### Sanger sequencing

The three identified variants in chr34 (Table 1.) were genotyped by standard PCR and Sanger sequencing. Gene-specific primers were designed using Primer3 [66]. The PCR primers used in genotyping were: *LAMP3* forward 5’-ATGATTCGCGTCTTAGGTGGA-3’ and reverse 5’-TAAAAGTCAAGCCCGGTTGT-3’; *ABCC5* forward 5’-GCCTTTTCTTGCTGAGTTCATG-3’ and reverse 5’-TCAACCACCAATTGCTGAAGG-3’; *KLHL6* forward 5’-TGAATGCCCACTGTGTTTCC-3’ and reverse 5’-AGAACACACTCCTCCGTCAG-3’. Biotools DNA Polymerase was used for the amplification and the PCR products were purified using Exonuclease I (20U/μl, Thermo Fischer Scientific) and FastAP (1 U/ μl, Thermo Fischer Scientific). The PCR products were sequenced with forward primer using ABI 3730 capillary sequencer (Applied Biosystems, Life Technologies) at the Institute for Molecular Medicine Finland core facility (FIMM, Technology Centre, University of Helsinki, Helsinki, Finland). Sequencher 5.1 (GeneCodes) was used to analyze the Sanger sequence data.

### Large-scale variant screening

Additional screening for the *LAMP3* variant was carried out in a diverse sample set of dogs representing 297 breeds (S7 Table) submitted for routine commercial screening at Genoscoper Laboratories Oy, Helsinki, during 2018–2019. Genotyping was carried out according to manufacturer-recommended standard protocols on a custom designed Illumina Infinum XT genotyping bead chip (Illumina, San Diego, CA, USA) commercially available as the MyDogDNA / Optimal Selection panel test [67,68].

## Supporting information

S1 Table Coordinates and p-values of the 198 genome-wide significant SNPs. (XLSX)

S2 Table Summary statistics for the WES data. (DOCX)

S3 Table A list of publicly available whole genome sequencing data that was used for filtering. (DOCX)

S4 Table Metadata of the 887 control genomes used in the WGS variant filtering. (XLSX)

S5 Table Results of the whole genome filtering of the affected dog. (XLSX)

S6 Table Alignment of the canine LAMP3 protein sequence with 35 other species. (DOCX)

S7 Table Prevalence of the LAMP3 g.16,092,728C>T variant in 6940 dogs from 279 breeds and breed variants. (XLSX)

S1 Fig The QQ plot of the GWAS results. Raw (p_raw_) and genomic control corrected (p_GC_) p-values are denoted with gray and black, respectively. The lambda of the raw p-values (1.11) and the deviation from the diagonal indicate slight inflation in the cohort.
(PNG)

## References

[1] Fehrenbach H. Alveolar epithelial type II cell: Defender of the alveolus revisited. Respir. Res. 2001;2: 33–46. 10.1186/rr36 11686863 PMC59567

[2] Ochs M. The closer we look the more we see? Quantitative microscopic analysis of the pulmonary surfactant system. Cell Physiol Biochem. 2010;25: 27–40. 10.1159/000272061 20054142

[3] Dietl P, Haller T, Mair N, Frick M. Mechanisms of Surfactant Exocytosis in Alveolar Type II Cells In Vitro and In Vivo. Physiology. 2001;16: 239. 10.1152/physiologyonline.2001.16.5.239 11572929

[4] Dell'Angelica EC, Mullins C, Caplan S, Bonifacino JS. Lysosome-related organelles. FASEB J. 2000;14: 1265–1278. 10.1096/fj.14.10.1265 10877819

[5] Weaver TE, Na C, Stahlman M. Biogenesis of lamellar bodies, lysosome-related organelles involved in storage and secretion of pulmonary surfactant. Semin. Cell Dev. Biol. 2002;13: 263–270. 10.1016/s1084952102000551 12243725

[6] Nogee LM. Interstitial lung disease in newborns. Semin Fetal Neonatal Med. 2017;22: 227–233. 10.1016/j.siny.2017.03.003 28363760 PMC5537026

[7] Shulenin S, Nogee LM, Annilo T, Wert SE, Whitsett JA, Dean M. ABCA3 Gene Mutations in Newborns with Fatal Surfactant Deficiency. N. Engl. J. Med. 2004;350: 1296–1303. 10.1056/NEJMoa032178 15044640

[8] Nogee LM, de Mello DE, Dehner LP, Colten HR. Brief report: Genome Sequence protein B in congenital alveolar proteinosis. N. Engl. J. Med. 1993;328: 406–410. 10.1056/NEJM199302113280606 8421459

[9] Nogee LM, Dunbar AE, Wert SE, Askin F, Hamvas A, Whitsett JA. A Mutation in the Surfactant Protein C Gene Associated with Familial Interstitial Lung Disease. N Engl J Med. 2001;344: 573–579. 10.1056/NEJM200102223440805 11207353

[10] Lindblad-Toh K, Wade CM, Mikkelsen TS, Karlsson EK, Jaffe DB, Kamal M, et al Genome sequence, comparative analysis and haplotype structure of the domestic dog. Nature. 2005;438: 803–819. 10.1038/nature04338 16341006

[11] Hytönen MK, Lohi H. Canine models of human rare disorders. Rare Diseases. 2016;4: e1241362 10.1080/21675511.2016.1241362 27803843 PMC5070630

[12] Wielaender F, Sarviaho R, James F, Hytonen MK, Cortez MA, Kluger G, et al Generalized myoclonic epilepsy with photosensitivity in juvenile dogs caused by a defective DIRAS family GTPase 1. Proc Natl Acad Sci U S A. 2017;114: 2669–2674. 10.1073/pnas.1614478114 28223533 PMC5347561

[13] Kaukonen M, Woods S, Ahonen S, Lemberg S, Hellman M, Hytönen MK, et al Maternal Inheritance of a Recessive RBP4 Defect in Canine Congenital Eye Disease. Cell Reports. 2018;23: 2643–2652. 10.1016/j.celrep.2018.04.118 29847795 PMC6546432

[14] Holopainen S, Hytönen MK, Syrjä P, Arumilli M, Järvinen A, Rajamäki M, et al ANLN truncation causes a familial fatal acute respiratory distress syndrome in Dalmatian dogs. PLoS genetics. 2017;13: e1006625 10.1371/journal.pgen.1006625 28222102 PMC5340406

[15] Hug P, Anderegg L, Kehl A, Jagannathan V, Leeb T. AKNA Frameshift Variant in Three Dogs with Recurrent Inflammatory Pulmonary Disease. Genes. 2019;10: 567 10.3390/genes10080567 31357536 PMC6723478

[16] Anderegg L, Im Hof Gut M, Hetzel U, Howerth EW, Leuthard F, Kyöstilä K, et al NME5 frameshift variant in Alaskan Malamutes with primary ciliary dyskinesia. PLoS genetics. 2019;15: e1008378 10.1371/journal.pgen.1008378 31479451 PMC6743793

[17] Saint-Vis B, Vincent J, Vandenabeele S, Vanbervliet B, Pin J-, Aït-Yahia S, et al A Novel Lysosome-Associated Membrane Glycoprotein, DC-LAMP, Induced upon DC Maturation, Is Transiently Expressed in MHC Class II Compartment. Immunity. 1998;9: 325–336. 10.1016/s1074-7613(00)80615-9 9768752

[18] Akasaki K, Nakamura N, Tsukui N, Yokota S, Murata S, Katoh R, et al Human dendritic cell lysosome-associated membrane protein expressed in lung type II pneumocytes. Arch. Biochem. Biophys. 2004;425: 147–157. 10.1016/j.abb.2004.02.042 15111122

[19] Salaun B, de Saint-Vis B, Pacheco Y, Pacheco N, Riesler A, Isaac S, et al CD208/Dendritic Cell-Lysosomal Associated Membrane Protein Is a Marker of Normal and Transformed Type II Pneumocytes. Am. J. Pathol. 2004;164: 861–871. 10.1016/S0002-9440(10)63174-4 14982840 PMC1613301

[20] Lewin G, Hurtt ME. Pre- and Postnatal Lung Development: An Updated Species Comparison. Birth Defects Res. 2017;109: 1519–1539. 10.1002/bdr2.1089 28876535

[21] Bertocci B, Lecoeuche D, Sterlin D, Kuhn J, Gaillard B, De Smet A, et al Klhl6 Deficiency Impairs Transitional B Cell Survival and Differentiation. J. Immunol. 2017;199: 2408–2420. 10.4049/jimmunol.1700708 28807996

[22] Jansen RS, Mahakena S, de Haas M, Borst P, van de Wetering K. ATP-binding cassette subfamily C member 5 (ABCC5) functions as an efflux transporter of glutamate conjugates and analogs. J. Biol. Chem. 2015;290: 30429–30440. 10.1074/jbc.M115.692103 26515061 PMC4683265

[23] Wolf De, Cornelia J. F, Yamaguchi H, Van Der Heijden I, Wielinga PR, Hundscheid SL, Ono N, et al cGMP transport by vesicles from human and mouse erythrocytes. FEBS Journal. 2007;274: 439–450. 10.1111/j.1742-4658.2006.05591.x 17229149

[24] Yamano G, Funahashi H, Kawanami O, Zhao L, Ban N, Uchida Y, et al ABCA3 is a lamellar body membrane protein in human lung alveolar type II cells. FEBS Lett. 2001;508: 221–225. 10.1016/s0014-5793(01)03056-3 11718719

[25] Choi Y, Chan AP. PROVEAN web server: a tool to predict the functional effect of amino acid substitutions and indels. Bioinformatics. 2015;31: 2745–2747. 10.1093/bioinformatics/btv195 25851949 PMC4528627

[26] Bendl J, Stourac J, Salanda O, Pavelka A, Wieben ED, Zendulka J, et al PredictSNP: robust and accurate consensus classifier for prediction of disease-related mutations. PLoS Comput. Biol. 2014;10: e1003440 10.1371/journal.pcbi.1003440 24453961 PMC3894168

[27] Sievers F, Wilm A, Dineen D, Gibson TJ, Karplus K, Li W, et al Fast, scalable generation of high‐quality protein multiple sequence alignments using Clustal Omega. Mol. Syst. Biol. 2011;7: 539–n/a. 10.1038/msb.2011.75 21988835 PMC3261699

[28] Chen R, Shi L, Hakenberg J, Naughton B, Sklar P, Zhang J, et al Analysis of 589,306 genomes identifies individuals resilient to severe Mendelian childhood diseases. Nature biotechnology. 2016;34: 531–538. 10.1038/nbt.3514 27065010

[29] Vieira NM, Elvers I, Alexander MS, Moreira YB, Eran A, Gomes JP, et al Jagged 1 Rescues the Duchenne Muscular Dystrophy Phenotype. Cell. 2015;163: 1204–1213. 10.1016/j.cell.2015.10.049 26582133 PMC4668935

[30] Szafranski P, Liu Q, Karolak J, Song X, de Leeuw N, Faas B, et al Association of rare non-coding SNVs in the lung-specific FOXF1 enhancer with a mitigation of the lethal ACDMPV phenotype. Hum Genet. 2019;138: 1301–1311. 10.1007/s00439-019-02073-x 31686214 PMC6874894

[31] Arboleda-Velasquez JF, Lopera F, O’Hare M, Delgado-Tirado S, Marino C, Chmielewska N, et al Resistance to autosomal dominant Alzheimer’s disease in an APOE3 Christchurch homozygote: a case report. Nature Medicine. 2019;25: 1680–1683. 10.1038/s41591-019-0611-3 31686034 PMC6898984

[32] Witschi H. Proliferation of type II alveolar cells: A review of common responses in toxic lung injury. Toxicology. 1976;5: 267–277. 10.1016/0300-483x(76)90046-9 817421

[33] Barkauskas CE, Cronce MJ, Rackley CR, Bowie EJ, Keene DR, Stripp BR, et al Type 2 alveolar cells are stem cells in adult lung. J. Clin. Invest. 2013;123: 3025 10.1172/JCI68782 23921127 PMC3696553

[34] Ochs M, Nenadic I, Fehrenbach A, Albes JM, Wahlers T, Richter J, et al Ultrastructural alterations in intraalveolar surfactant subtypes after experimental ischemia and reperfusion. Am. J. Respir. Crit. Care Med. 1999;160: 718–724. 10.1164/ajrccm.160.2.9809060 10430751

[35] Ochs M, Fehrenbach H, Richter J. Ultrastructure of canine type II pneumocytes during hypothermic ischemia of the lung: A study by means of conventional and energy filtering transmission electron microscopy and stereology. Anat. Rec. 2001;263: 118–126. 10.1002/ar.1084 11360229

[36] Edwards V, Cutz E, Viero S, Moore AM, Nogee L. Ultrastructure of Lamellar Bodies in Congenital Surfactant Deficiency. Ultrastruct. Pathol. 2005;29: 503–509. 10.1080/01913120500323480 16316951

[37] Stahlman MT, Phillips Gray M, Falconieri MW, Whitsett JA, Weaver TE. Lamellar Body Formation in Normal and Surfactant Protein B-Deficient Fetal Mice. Lab. Investig. 2000;80: 395–403. 10.1038/labinvest.3780044 10744075

[38] Citti A, Peca D, Petrini S, Cutrera R, Biban P, Haass C, et al Ultrastructural Characterization of Genetic Diffuse Lung Diseases in Infants and Children: A Cohort Study and Review. Ultrastruct. Pathol. 2013;37: 356–365. 10.3109/01913123.2013.811454 24047351

[39] Glasser ST, Burhans MS, Korfhagen TR, Na C, Sly PD, Ross GF, et al Altered Stability of Pulmonary Surfactant in SP-C-Deficient Mice. Proc. Natl. Acad. Sci. U.S.A. 2001;98: 6366–6371. 10.1073/pnas.101500298 11344267 PMC33474

[40] Liao X, Song L, Zhang L, Wang H, Tong Q, Xu J, et al LAMP3 regulates hepatic lipid metabolism through activating PI3K/Akt pathway. Molecular and Cellular Endocrinology. 2018;470: 160–167. 10.1016/j.mce.2017.10.010 29056532

[41] Kanao H, Enomoto T, Kimura T, Fujita M, Nakashima R, Ueda Y, et al Overexpression of LAMP3/TSC403/DC-LAMP Promotes Metastasis in Uterine Cervical Cancer. Cancer Research. 2005;65: 8640 10.1158/0008-5472.CAN-04-4112 16204031

[42] Wilke S, Krausze J, Büssow K. Crystal structure of the conserved domain of the DC lysosomal associated membrane protein: implications for the lysosomal glycocalyx. BMC Biol. 2012;10: 62 10.1186/1741-7007-10-62 22809326 PMC3409847

[43] Terasawa K, Tomabechi Y, Ikeda M, Ehara H, Kukimoto-Niino M, Wakiyama M, et al Lysosome-associated membrane proteins-1 and -2 (LAMP-1 and LAMP-2) assemble via distinct modes. Biochem. Biophys. Res. Commun. 2016;479: 489–495. 10.1016/j.bbrc.2016.09.093 27663661

[44] Engelbrecht S, Kaltenborn E, Griese M, Kern S. The surfactant lipid transporter ABCA3 is N-terminally cleaved inside LAMP3-positive vesicles. FEBS Lett. 2010;584: 4306–4312. 10.1016/j.febslet.2010.09.026 20863830

[45] Brasch F, Ochs M, Kahne T, Guttentag S, Schauer-Vukasinovic V, Derrick M, et al Involvement of Napsin A in the C- and N-terminal Processing of Surfactant Protein B in Type-II Pneumocytes of the Human Lung. J. Biol. Chem. 2003;278: 49006–49014. 10.1074/jbc.M306844200 13129928

[46] Johnson AL, Braidotti P, Pietra GG, Russo SJ, Kabore A, Wang W, et al Post-Translational Processing of Surfactant Protein-C Proprotein. Targeting Motifs in the NH2-Terminal Flanking Domain Are Cleaved in Late Compartments. Am. J. Respir. Cell Mol. Biol. 2001;24: 253–263. 10.1165/ajrcmb.24.3.4312 11245624

[47] Cheong N, Muniswamy Madesh, Linda W. Gonzales, Ming Zhao, Kevin Yu, Philip L. Ballard, et al Functional and Trafficking Defects in ATP Binding Cassette A3 Mutants Associated with Respiratory Distress Syndrome. J. Biol. Chem. 2006;281: 9791–9800. 10.1074/jbc.M507515200 16415354

[48] Ban N, Yoshihiro M, Hiromichi S, Yasukazu T, Mayumi S, Hiroyuki A, et al ABCA3 as a Lipid Transporter in Pulmonary Surfactant Biogenesis. J. Biol. Chem. 2007;282: 9628–9634. 10.1074/jbc.M611767200 17267394

[49] Wittmann T, Schindlbeck U, Höppner S, Kinting S, Frixel S, Kröner C, et al Tools to explore ABCA3 mutations causing interstitial lung disease. Pediatr. Pulmonol. 2016;51: 1284–1294. 10.1002/ppul.23471 27177387

[50] Mandrile G, Dubois A, Hoffman JD, Uliana V, Di Maria E, Malacarne M, et al 3q26.33–3q27.2 microdeletion: A new microdeletion syndrome? Eur J Med Genet. 2013;56: 216–221. 10.1016/j.ejmg.2013.01.005 23357683

[51] Dasouki M, Roberts J, Santiago A, Saadi I, Hovanes K. Confirmation and further delineation of the 3q26.33–3q27.2 microdeletion syndrome. Eur J Med Genet. 2014;57: 76–80. 10.1016/j.ejmg.2013.12.007 24462885

[52] Somaschi M, Nogee LM, Sassi I, Danhaive O, Presi S, Boldrini R, et al Unexplained Neonatal Respiratory Distress Due to Congenital Surfactant Deficiency. J. Pediatr. 2007;150: 649–653.e1. 10.1016/j.jpeds.2007.03.008 17517255

[53] Kennelliitto Suomen [Internet]. Koiranet Jalostustietojärjestelmä [cited 2018 Nov 8]. 2018 Available from: https://jalostus.kennelliitto.fi/.

[54] Karnovsky MJ. Use of ferrocyanide-reduced osmium tetroxide in electron microscopy. Proc. 11th Ann.Meeting. Am Soc Cell Biol. 1971: 146a.

[55] Purcell S, Neale B, Todd-Brown K, Thomas L, Ferreira MA, Bender D, et al PLINK: a tool set for whole-genome association and population-based linkage analyses. Am J Hum Genet. 2007;81: 559–575. S0002-9297(07)61352-4 [pii]. 10.1086/519795 17701901 PMC1950838

[56] Broeckx BJ, Hitte C, Coopman F, Verhoeven GE, De Keulenaer S, De Meester E, et al Improved canine exome designs, featuring ncRNAs and increased coverage of protein coding genes. Sci Rep. 2015;5: 12810 10.1038/srep12810 26235384 PMC4522663

[57] Li H, Durbin R. Fast and accurate short read alignment with Burrows-Wheeler transform. Bioinformatics. 2009;25: 1754–1760. 10.1093/bioinformatics/btp324 19451168 PMC2705234

[58] Broad Institute Github repository [Internet]. Picard Toolkit [cited 2018 Nov 20]. 2018. Available from: http://broadinstitute.github.io/picard/.

[59] McKenna A, Hanna M, Banks E, Sivachenko A, Cibulskis K, Kernytsky A, et al The Genome Analysis Toolkit: a MapReduce framework for analyzing next-generation DNA sequencing data. Genome Res. 2010;20: 1297–1303. 10.1101/gr.107524.110 20644199 PMC2928508

[60] Layer RM, Kindlon N, Karczewski KJ, Exome Aggregation Consortium, Quinlan AR. Efficient genotype compression and analysis of large genetic-variation data sets. Nat Methods. 2016;13: 63–65. 10.1038/nmeth.3654 26550772 PMC4697868

[61] Arumilli M, Layer R, Hytönen M, Lohi H. webGQT: A Graphical User Interface for Genotype Query Tools, Front Genet, Forthcoming 2020. 10.3389/fgene.2020.00152 PMC7063093 32194629

[62] Rausch T, Zichner T, Schlattl A, Stütz AM, Benes V, Korbel JO. DELLY: structural variant discovery by integrated paired-end and split-read analysis. Bioinformatics (Oxford, England). 2012;28: i333–i339. 10.1093/bioinformatics/bts378 22962449 PMC3436805

[63] Bao W, Kojima KK, Kohany O. Repbase Update, a database of repetitive elements in eukaryotic genomes. Mobile DNA. 2015;6: 11 10.1186/s13100-015-0041-9 26045719 PMC4455052

[64] Gardner EJ, Lam VK, Harris DN, Chuang NT, Scott EC, Pittard WS, et al The Mobile Element Locator Tool (MELT): population-scale mobile element discovery and biology. Genome research. 2017;27: 1916–1929. 10.1101/gr.218032.116 28855259 PMC5668948

[65] Thongtharb A, Uchida K, Chambers JK, Kagawa Y, Nakayama H. Histological and immunohistochemical studies on primary intracranial canine histiocytic sarcomas. J. Vet. Med. Sci. 2016;78: 593–599. 10.1292/jvms.15-0627 26668164 PMC4873849

[66] Untergasser A, Cutcutache I, Koressaar T, Ye J, Faircloth BC, Remm M, et al Primer3-new capabilities and interfaces. Nucleic Acids Res. 2012;40: e115 10.1093/nar/gks596 22730293 PMC3424584

[67] Donner J, Kaukonen M, Anderson H, Möller F, Kyöstilä K, Sankari S, et al Genetic Panel Screening of Nearly 100 Mutations Reveals New Insights into the Breed Distribution of Risk Variants for Canine Hereditary Disorders. PloS one. 2016;11: e0161005 10.1371/journal.pone.0161005 27525650 PMC4985128

[68] Donner J, Anderson H, Davison S, Hughes AM, Bouirmane J, Lindqvist J, et al Frequency and distribution of 152 genetic disease variants in over 100,000 mixed breed and purebred dogs. PLoS genetics. 2018;14: e1007361 10.1371/journal.pgen.1007361 29708978 PMC5945203

