# Supplementary material for: Recessive missense LAMP3 variant associated with defect in lamellar body biogenesis and fatal neonatal interstitial lung disease in dogs
Source: PLoS Genet. 2020 Mar 9;16(3):e1008651. doi: 10.1371/journal.pgen.1008651 (PMC7082050; doi:10.1371/journal.pgen.1008651)
Supplement: S2 Table — (DOCX) [file pgen.1008651.s002.docx]

**S2 Table.** **Summary statistics of the whole exome sequencing data.**

| Sample | Status | Total reads | Mapped reads (%) | Mean coverage | Total variants | Homozygous variants |
| --- | --- | --- | --- | --- | --- | --- |
| AT324 | affected | 74,983,087 | 74,716,348 (99.64%) | 50.25 | 241814 | 136193 |
| AT330 | affected | 64,132,154 | 63,846,900 (99.56%) | 44.01 | 237190 | 138607 |
| AT008 | carrier | 78,379,956 | 78,054,493 (99.58%) | 53.26 | 245892 | 142521 |
