# Supplementary material for: Recessive missense LAMP3 variant associated with defect in lamellar body biogenesis and fatal neonatal interstitial lung disease in dogs
Source: PLoS Genet. 2020 Mar 9;16(3):e1008651. doi: 10.1371/journal.pgen.1008651 (PMC7082050; doi:10.1371/journal.pgen.1008651)
Supplement: S3 Table — (DOCX) [file pgen.1008651.s003.docx]

**S3 Table.** A list of publicly available whole genome sequencing data that was used for filtering.

| **Breed** | **N** | **Study accession number** |
| --- | --- | --- |
| Airedale Terrier | 1 | PRJEB16012 |
| Alaskan Husky (not purebred) | 1 | PRJEB9590 |
| Alaskan Malamute | 1 | PRJEB16012 |
| Alpine Dachsbracke | 1 | PRJEB14840 |
| American Staffordshire Terrier | 1 | PRJEB16012 |
| Australian Cattle Dog | 3 | PRJEB16012; PRJEB13468 |
| Australian Terrier | 1 | PRJEB16012 |
| Basset Hound | 1 | PRJEB16012 |
| Bavarian Hound | 1 | PRJEB16012 |
| Beagle | 1 | PRJEB5500 |
| Bearded Collie | 8 | PRJEB13468; PRJEB16012 |
| Belgian Shepherd Dog | 6 | PRJEB16012; PRJNA319610; SRP074053 |
| Border Collie | 13 | PRJEB4544 |
| Bull Terrier | 1 | PRJEB16012 |
| Bullmastiff | 1 | PRJEB16012 |
| Cavalier King Charles Spaniel | 2 | PRJEB16012 |
| Central Asian Shepherd Dog | 1 | PRJNA360671; SRP096308 |
| Chihuahua | 1 | PRJEB13139 |
| Chinese indigenous dog | 38 | PRJNA266585 |
| Chow Chow | 1 | PRJEB16012 |
| Cocker Spaniel | 1 | PRJEB16012 |
| Dachshund | 3 | PRJEB16012; PRJEB7736 |
| Dalmatian | 1 | PRJNA360671; SRP096308 |
| Dobermann | 3 | PRJEB16012 |
| Elo | 1 | PRJEB16012 |
| Entelbuch Cattle Dog | 8 | PRJEB16012 |
| Eurasier | 2 | PRJEB6079; PRJEB16012 |
| French Bulldog | 2 | PRJEB13468; PRJEB16012 |
| German Hunting Terrier | 1 | PRJEB16012 |
| German Pointing Dog | 1 | PRJEB13468 |
| German Shepherd Dog | 13 | PRJEB16012; PRJNA266585 |
| German Shepherd Dog (mixed breed) | 3 | PRJEB14110 |
| Golden Retriever | 4 | PRJEB16012 |
| Golden Retriever (mixed breed) | 1 | PRJEB16012 |
| Great Dane | 1 | PRJEB16012 |
| Greater Swiss Mountain Dog | 1 | PRJEB16012 |
| Greyhound | 1 | PRJEB16012 |
| Grosspitz | 1 | PRJEB16012 |
| Heideterrier | 1 | PRJEB16012 |
| Hovawart | 1 | PRJEB16012 |
| Irish Soft Coated Wheaten Terrier | 1 | PRJNA421138 |
| Irish Terrier | 1 | PRJEB13468 |
| Jack Russell Terrier | 1 | PRJEB16012 |
| Kromfohrländer | 1 | PRJEB6076 |
| Labrador Retriever | 4 | PRJEB16012; PRJEB5874; PRJEB5875 |
| Lagotto Romagnolo | 5 | PRJEB16012; PRJEB13468 |
| Landseer | 2 | PRJEB9437; PRJEB7734 |
| Leonberger | 2 | PRJEB16012 |
| Miniature Bullterrier | 1 | PRJEB16012 |
| Mixed Breed | 2 | PRJEB16012 |
| Norwich Terrier | 4 | PRJEB16012 |
| Old English Sheepdog | 1 | PRJEB16012 |
| Pomeranian | 1 | PRJEB16012 |
| Rhodesian Ridgeback | 4 | PRJEB16012; PRJNA357866; SRP095269 |
| Rottweiler | 1 | PRJEB7735 |
| Saluki | 1 | PRJEB16012 |
| Shetland Sheepdog | 1 | PRJEB16012 |
| Siberian Husky | 3 | PRJEB10823; PRJEB9591; PRJEB16012 |
| Sloughi | 3 | PRJEB13468; PRJEB16012 |
| Spanish Waterdog | 1 | PRJEB7903 |
| Swedish Vallhund | 2 | PRJNA394814; SRP112685 |
| Tibetan Mastiff | 10 | PRJNA266585 |
| Weimaraner | 1 | PRJEB16012 |
| West Highland White Terrier | 7 | PRJEB16012; PRJEB13723 |
| Whippet | 1 | PRJEB13468 |
| White Swiss Shepherd Dog | 1 | PRJEB16012 |
| Wolf | 3 | PRJEB16012 |
| Yorkshire Terrier | 1 | PRJEB13468 |
|  | 200 |  |
