# Supplementary material for: Recessive missense LAMP3 variant associated with defect in lamellar body biogenesis and fatal neonatal interstitial lung disease in dogs
Source: PLoS Genet. 2020 Mar 9;16(3):e1008651. doi: 10.1371/journal.pgen.1008651 (PMC7082050; doi:10.1371/journal.pgen.1008651)

**S6 Table.** **Alignment of the canine LAMP3 protein sequence with 35 other species.** The p.E387K variant position highlighted in blue.


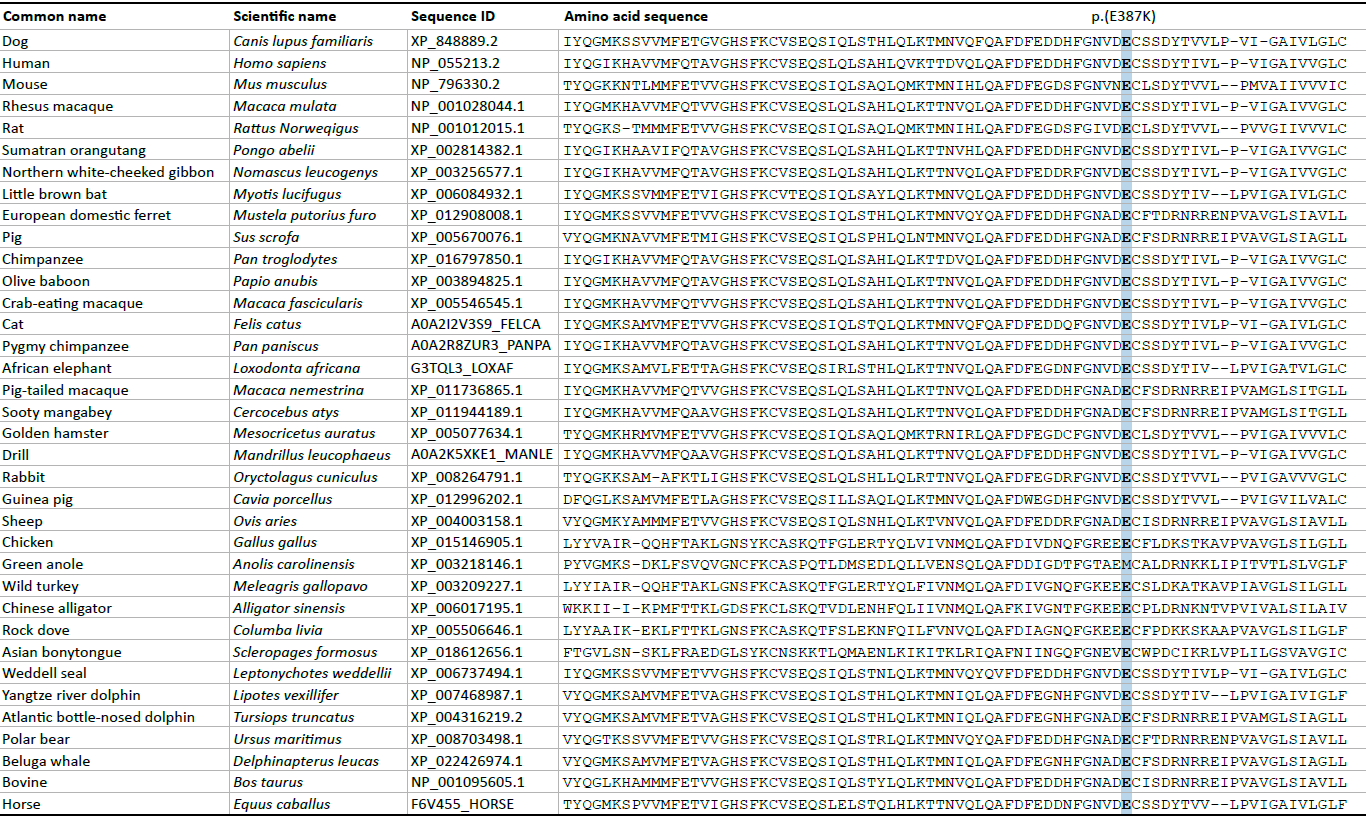

Supplement: S6 Table — (DOCX) [file pgen.1008651.s006.docx]
