# Supplementary figures and images for: Recessive missense LAMP3 variant associated with defect in lamellar body biogenesis and fatal neonatal interstitial lung disease in dogs
Source: PLoS Genet. 2020 Mar 9;16(3):e1008651. doi: 10.1371/journal.pgen.1008651 (PMC7082050; doi:10.1371/journal.pgen.1008651)

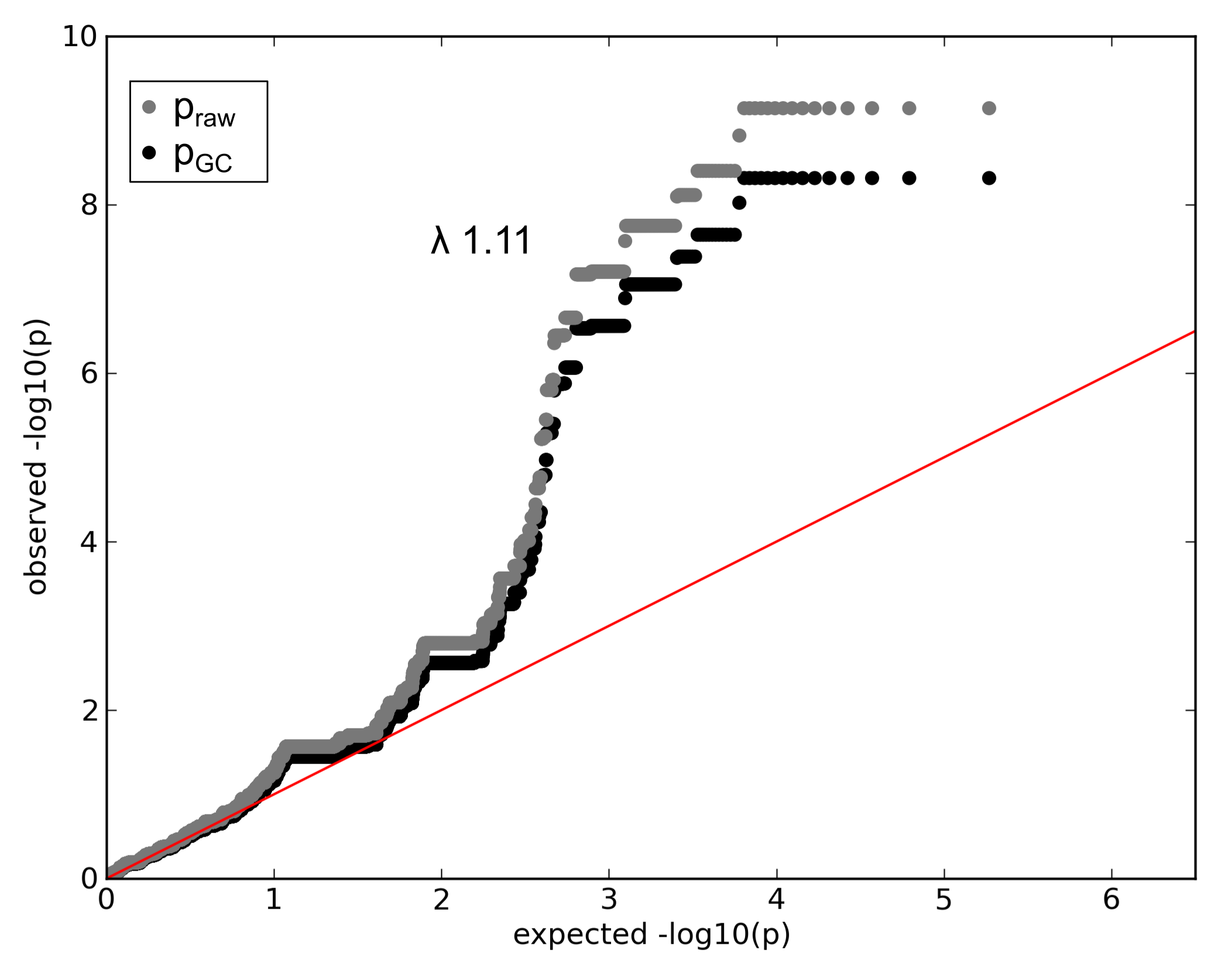

Supplement: S1 Fig — Raw (praw) and genomic control corrected (pGC) p-values are denoted with gray and black, respectively. The lambda of the raw p-values (1.11) and the deviation from the diagonal indicate slight inflation in the cohort. (PNG) [file pgen.1008651.s008.png]
